# Retraction: Targeting and Therapy of Glioblastoma in a Mouse Model Using Exosomes Derived From Natural Killer Cells

**DOI:** 10.3389/fimmu.2019.01770

**Published:** 2019-07-16

**Authors:** Frontiers Editorial Office

The journal and Chief Editors retract the April 2018 article cited above. Following the publication of the article, concerns regarding the results were addressed to the journal and its Editorial Board. Following clarification with the authors, errors were identified with the data and the article is therefore retracted with the agreement of the authors.

